# Supplementary material for: End Sequence Analysis Toolkit (ESAT) expands the extractable information from single-cell RNA-seq data
Source: Genome Res. 2016 Oct;26(10):1397–410. doi: 10.1101/gr.207902.116 (PMC5052061; doi:10.1101/gr.207902.116)
Supplement: Supplemental Material [file supp_gr.207902.116_Supp_Materials.tar.gz › ESAT-esat_v0.1/doc/html/classbroad_1_1pda_1_1seq_1_1segmentation_1_1_alignment_data_model_stats-members.html]

ESAT: Member List


ESAT


- Main Page
- Related Pages
- Packages
- Classes
- Files

- Class List
- Class Index
- Class Hierarchy
- Class Members

All Classes Namespaces Files Functions Variables Enumerator Pages

- broad
- pda
- seq
- segmentation
- AlignmentDataModelStats

broad.pda.seq.segmentation.AlignmentDataModelStats Member List

This is the complete list of members for broad.pda.seq.segmentation.AlignmentDataModelStats, including all inherited members.

|  |  |  |
| --- | --- | --- |
| AlignmentDataModelStats() | broad.pda.seq.segmentation.AlignmentDataModelStats |  |
| AlignmentDataModelStats(AlignmentDataModel data, Map< String, Integer > maskedRegions, String chr) | broad.pda.seq.segmentation.AlignmentDataModelStats |  |
| AlignmentDataModelStats(AlignmentDataModel data, Map< String, Integer > maskedRegions, String chr, double lambda, boolean loadChromosomeStats) | broad.pda.seq.segmentation.AlignmentDataModelStats |  |
| AlignmentDataModelStats(AlignmentDataModel data, Map< String, Integer > maskedRegions) | broad.pda.seq.segmentation.AlignmentDataModelStats |  |
| AlignmentDataModelStats(AlignmentDataModel data, Map< String, Integer > maskedRegions, Map< String, Double > readsPerChromosome) | broad.pda.seq.segmentation.AlignmentDataModelStats |  |
| AlignmentDataModelStats(AlignmentDataModel data, Map< String, Integer > maskedRegions, String chr, boolean loadChromosomeStats) | broad.pda.seq.segmentation.AlignmentDataModelStats |  |
| AlignmentDataModelStats(AlignmentDataModel data, Map< String, Integer > maskedRegions, double lambda) | broad.pda.seq.segmentation.AlignmentDataModelStats |  |
| AlignmentDataModelStats(AlignmentDataModel data, Map< String, Integer > maskedRegions, double lambda, boolean loadChromosomeStats) | broad.pda.seq.segmentation.AlignmentDataModelStats |  |
| AlignmentDataModelStats(AlignmentDataModel data, Map< String, Integer > maskedRegions, String chr, double lambda) | broad.pda.seq.segmentation.AlignmentDataModelStats |  |
| AlignmentDataModelStats(AlignmentDataModel data, String chr) | broad.pda.seq.segmentation.AlignmentDataModelStats |  |
| AlignmentDataModelStats(AlignmentDataModel data) | broad.pda.seq.segmentation.AlignmentDataModelStats |  |
| chromosomeLength(String chr) | broad.pda.seq.segmentation.AlignmentDataModelStats |  |
| count(Annotation align, int extensionFactor) | broad.pda.seq.segmentation.AlignmentDataModelStats |  |
| countWithinExon(Annotation align, int extensionFactor) | broad.pda.seq.segmentation.AlignmentDataModelStats |  |
| getAnnotationOverlappingRegion(Annotation Annotation) | broad.pda.seq.segmentation.AlignmentDataModelStats |  |
| getChromosomeLengths() | broad.pda.seq.segmentation.AlignmentDataModelStats |  |
| getChunkEnd() | broad.pda.seq.segmentation.AlignmentDataModelStats |  |
| getChunkStart() | broad.pda.seq.segmentation.AlignmentDataModelStats |  |
| getCounts(Collection< Annotation > Annotation, int extensionFactor) | broad.pda.seq.segmentation.AlignmentDataModelStats |  |
| getCountsOfUniqueOverlappers(Annotation startPosition, Annotation window, IntervalTree< Alignment > chunkAlignmentTree, int extensionFactor) | broad.pda.seq.segmentation.AlignmentDataModelStats |  |
| getCountsPerAlignment(Annotation align, IntervalTree< Alignment > tree, int extensionFactor) | broad.pda.seq.segmentation.AlignmentDataModelStats |  |
| getCountsPerAlignment(Gene window, IntervalTree< Alignment > tree, int extensionFactor) | broad.pda.seq.segmentation.AlignmentDataModelStats |  |
| getCountsPerAlignment(Annotation align, Map< String, IntervalTree< Annotation >> exonTree, IntervalTree< Alignment > tree, int extensionFactor) | broad.pda.seq.segmentation.AlignmentDataModelStats |  |
| getData() | broad.pda.seq.segmentation.AlignmentDataModelStats |  |
| getExonAnnotationOverlappingRegion(Annotation align) | broad.pda.seq.segmentation.AlignmentDataModelStats |  |
| getGeneCounts(Collection< Gene > genes, int extensionFactor) | broad.pda.seq.segmentation.AlignmentDataModelStats |  |
| getGeneCountsWithoutDatasetTotals(Collection< Gene > genes, int extensionFactor) | broad.pda.seq.segmentation.AlignmentDataModelStats |  |
| getGeneExpression(Collection< Gene > genes, int extensionFactor) | broad.pda.seq.segmentation.AlignmentDataModelStats |  |
| getGlobalRPKMConstant() | broad.pda.seq.segmentation.AlignmentDataModelStats |  |
| getIntervalTree(LightweightGenomicAnnotation region) | broad.pda.seq.segmentation.AlignmentDataModelStats |  |
| getIntervalTree(String chr, int start, int end) | broad.pda.seq.segmentation.AlignmentDataModelStats |  |
| getIntervalTreeCached(String chr, int start, int end) | broad.pda.seq.segmentation.AlignmentDataModelStats |  |
| getIntervalTreeTruncatedCached(String chromosome, int start, int end) | broad.pda.seq.segmentation.AlignmentDataModelStats |  |
| getLambda(String chr) | broad.pda.seq.segmentation.AlignmentDataModelStats |  |
| getMaskedRegionsSize(String chr) | broad.pda.seq.segmentation.AlignmentDataModelStats |  |
| getMinimumMappingQuality() | broad.pda.seq.segmentation.AlignmentDataModelStats |  |
| getNumberMarkers(String chr) | broad.pda.seq.segmentation.AlignmentDataModelStats |  |
| getNumberOfReads() | broad.pda.seq.segmentation.AlignmentDataModelStats |  |
| getNumberOfReads(String chr) | broad.pda.seq.segmentation.AlignmentDataModelStats |  |
| getNumberOfReadsByChr(String chr) | broad.pda.seq.segmentation.AlignmentDataModelStats |  |
| getPeak(Gene gene, Gene startCodon, IntervalTree< Alignment > tree, int EF) | broad.pda.seq.segmentation.AlignmentDataModelStats |  |
| getReadIterator() | broad.pda.seq.segmentation.AlignmentDataModelStats |  |
| getReadIterator(Annotation region) | broad.pda.seq.segmentation.AlignmentDataModelStats |  |
| getRPKMConstant(String chr) | broad.pda.seq.segmentation.AlignmentDataModelStats |  |
| getScorePerAlignmentFromCache(Gene window, IntervalTree< Alignment > chunkAlignmentTree, int extensionFactor) | broad.pda.seq.segmentation.AlignmentDataModelStats |  |
| getSplicedReads(Annotation chrRegion, int i, int j) | broad.pda.seq.segmentation.AlignmentDataModelStats |  |
| getSplicedReads(Annotation region, List< Predicate< Annotation >> filters, int minNumIntrons) | broad.pda.seq.segmentation.AlignmentDataModelStats |  |
| getSplicedReads(Annotation region, int minIntronSize, int maxIntronSize, int minNumberOfSplices, Sequence chrSequence) | broad.pda.seq.segmentation.AlignmentDataModelStats |  |
| getSpliceWeightFactor() | broad.pda.seq.segmentation.AlignmentDataModelStats |  |
| getSum(String chr) | broad.pda.seq.segmentation.AlignmentDataModelStats |  |
| getTotalReads() | broad.pda.seq.segmentation.AlignmentDataModelStats |  |
| init(Map< String, Integer > maskedRegions, double lambda, boolean loadChromosomeStats) | broad.pda.seq.segmentation.AlignmentDataModelStats |  |
| init(Map< String, Integer > maskedRegions, double lambda, boolean loadChromosomeStats, Map< String, Double > readsByChr) | broad.pda.seq.segmentation.AlignmentDataModelStats |  |
| isPaired() | broad.pda.seq.segmentation.AlignmentDataModelStats |  |
| normalizeReadCountsToBaseModel(AlignmentDataModelStats baseline) | broad.pda.seq.segmentation.AlignmentDataModelStats |  |
| passes(Gene window, IntervalTree< Alignment > chunkAlignmentTree, int i, int critVal) | broad.pda.seq.segmentation.AlignmentDataModelStats |  |
| resetTreeCache() | broad.pda.seq.segmentation.AlignmentDataModelStats |  |
| resetTreeCache(String chr) | broad.pda.seq.segmentation.AlignmentDataModelStats |  |
| scanPRate(Gene gene, int extensionFactor) | broad.pda.seq.segmentation.AlignmentDataModelStats |  |
| scanPRate(LightweightGenomicAnnotation annotation, int extensionFactor) | broad.pda.seq.segmentation.AlignmentDataModelStats |  |
| scanPRate(Gene gene, IntervalTree< Alignment > tree, int extensionFactor) | broad.pda.seq.segmentation.AlignmentDataModelStats |  |
| scanPRate(Annotation align, IntervalTree< Alignment > tree, double localLambda, int extensionFactor) | broad.pda.seq.segmentation.AlignmentDataModelStats |  |
| scanPRate(Annotation align, double readCount, int extenstionFactor) | broad.pda.seq.segmentation.AlignmentDataModelStats |  |
| scanPRate(Annotation align, double readCount, double localLambda, int extenstionFactor) | broad.pda.seq.segmentation.AlignmentDataModelStats |  |
| scanPRate(Gene gene, IntervalTree< Alignment > tree, double localLambda, int extensionFactor) | broad.pda.seq.segmentation.AlignmentDataModelStats |  |
| scanPRate(Gene gene, IntervalTree< Alignment > tree, double localLambda, int extensionFactor, double numMarkers) | broad.pda.seq.segmentation.AlignmentDataModelStats |  |
| scanRegion(int fixedWidth, LightweightGenomicAnnotation region) | broad.pda.seq.segmentation.AlignmentDataModelStats |  |
| setAlignmentDataModel(AlignmentDataModel data) | broad.pda.seq.segmentation.AlignmentDataModelStats |  |
| setChunkSize(int chunkSize) | broad.pda.seq.segmentation.AlignmentDataModelStats |  |
| setCountNormalizer(ReadCountNormalizer normalizer) | broad.pda.seq.segmentation.AlignmentDataModelStats |  |
| setExtensionFactor(int extensionFactor) | broad.pda.seq.segmentation.AlignmentDataModelStats |  |
| setLambda(String chr, double numberOfReads) | broad.pda.seq.segmentation.AlignmentDataModelStats |  |
| setMinimumMappingQuality(double minMapQual) | broad.pda.seq.segmentation.AlignmentDataModelStats |  |
| setNumberOfMarkers(String chr, double numMarkers2) | broad.pda.seq.segmentation.AlignmentDataModelStats |  |
| setNumberOfReads(String chr, double numberOfReads2) | broad.pda.seq.segmentation.AlignmentDataModelStats |  |
| setStoredStats(StoredAlignmentStats sas) | broad.pda.seq.segmentation.AlignmentDataModelStats |  |


---

Generated on Fri Aug 15 2014 15:11:04 for ESAT by  

 1.8.7
